# Impact of COVID-19 pandemic on surgical volume and outcomes in a terciary care center in Brazil

**DOI:** 10.1590/0100-6991e-20243678-en

**Published:** 2024-04-17

**Authors:** PAULO LISBOA BITTENCOURT, GABRIEL VIANNA PEREIRA ARAGÃO, MURILO TAVARES VALVERDE, GUILHERME ALMEIDA FARIAS AMORIM, IGOR LIMA VIEIRA DE CASTRO, JADE DE OLIVEIRA SANTANA, LAIANE CAITANO COSTA, BEATRIZ SOARES MARQUES MUNIZ, VIVIAN ROBERTA SOARES SILVA, LIANA CODES, CLAUDIO CELESTINO ZOLLINGER, WELLINGTON ANDRAUS

**Affiliations:** 1 - Hospital Português, Unidade de Gastroenterologia e Hepatologia - Salvador - BA - Brasil; 2 - Escola Bahiana de Medicina e Saúde Pública, Departamento de Gastroenterologia - Salvador - BA - Brasil; 3 - Universidade de São Paulo, Departamento de Gastroenterologia - São Paulo - Brasil

**Keywords:** Surgery, COVID-19, Mortality, Critical Care, Cirurgia Geral, COVID-19, Mortalidade, Cuidados Críticos

## Abstract

**Backgrounds:**

COVID-19 pandemic led to a sharp decline in surgical volume worldwide due to the postponement of elective procedures. This study evaluated the impact of COVID-19 pandemic in surgical volumes and outcomes of abdominal surgery in high-risk patients requiring intensive care unit admission.

**Methods:**

patients admitted for postoperative care were retrospectively evaluated. Data concerning perioperative variables and outcomes were compared in two different periods: January 2017-December 2019 and January 2020-December 2022, respectively, before (period I) and after (period II) the onset of COVID-19 pandemic.

**Results:**

1.402 patients (897 women, mean age 62+17 years) were investigated. Most of the patients underwent colorectal (n=393) and pancreato-biliary (n=240) surgery, 52% of elective procedures. Surgical volume was significantly lower in period II (n=514) when compared to period I (n= 888). No recovery was observed in the number of surgical procedures in 2022 (n=135) when compared to 2021(n=211) and 2020 (n=168). Subjects who underwent abdominal surgery in period II had higher Charlson comorbidity index (4,85+3,0 vs. 4,35+2,8, p=0,002), more emergent/urgent procedures (51% vs. 45%, p=0,03) and more clean-contaminated wounds (73,5% vs. 66,8%, p=0,02). A significant decrease in the volume of colorectal surgery was also observed (24% vs, 31%, p<0,0001) after the onset of COVID-19 pandemic, 125 (8,9%) died, no deaths due to COVID-19 infection. Mortality was higher in period II when compared to period I (11% vs. 8%, p=0,08).

**Conclusions:**

COVID-19 pandemic was associated with a decrease in surgical volume of high-risk patients without apparent recovery in recent years. No influence of COVID-19 was noted in postoperative mortality.

## RESUMO

**Introdução:**

a pandemia COVID-19 levou a um importante declínio mundial no volume cirúrgico devido ao adiamento de procedimentos eletivos. Este estudo avaliou o impacto da pandemia COVID-19 nos volumes e nos resultados da cirurgia abdominal em pacientes criticos.

**Métodos:**

pacientes internados para cuidados pósoperatórios foram avaliados retrospectivamente. Dados relativos aos desfechos perioperatórios foram comparados em dois períodos: janeiro-2017 a dezembro-2019 e janeiro-2020 a dezembro-2022, respectivamente, antes (período I) e depois (período II) da pandemia COVID-19.

**Resultados:**

foram investigados 1.402 pacientes (897 mulheres, idade 62+17 anos). A maioria dos pacientes foi submetida a cirurgia colorretal (n=393) e pancreatobiliar (n=240), sendo 52% dos procedimentos eletivos. O volume cirúrgico foi significativamente menor no período II (n=514) quando comparado ao período I (n=888). Não foi observada recuperação no número de procedimentos cirúrgicos em 2022 (n=135) quando comparado a 2021 (n=211) e 2020 (n=168). Indivíduos submetidos à cirurgia abdominal no período II apresentaram maior índice de comorbidade de Charlson (4,85+3,0 vs. 4,35+2,8, p=0,002), mais procedimentos emergenciais/urgentes (51% vs. 45%, p =0,03) e mais feridas potencialmente contaminadas (73,5% vs. 66,8%, p=0,02). Observou-se diminuição significativa no volume de cirurgia colorretal (24% vs, 31%, p<0,0001) após o início da pandemia de COVID-19, com 125 (8,9%) óbitos, nenhuma morte por COVID-19. A mortalidade foi maior no período II quando comparada ao período I (11% vs. 8%, p=0,08).

**Conclusões:**

a pandemia de COVID-19 foi associada à diminuição do volume cirúrgico de pacientes de alto risco sem recuperação aparente nos últimos anos. Nenhuma influência da COVID-19 foi observada na mortalidade pósoperatória.

## INTRODUCTION

COVID-19 is a highly contagious airborne viral disease caused by SARS-CoV2. The disease was initially reported in China spreading rapidly around the world to be declared by the World Health Organization (WHO) a global pandemic on March 11^th^, 2020^1^.Up to now according to WHO Coronavirus Dashboard, more than 770 million cases of SARS-COV2 infection were confirmed worldwide resulting in nearly7 million deaths around the globe^2^.

The disease course is usually with no respiratory or mild to moderate flu-like symptoms that may evolve in a smaller subset of the patients to pneumonia, acute respiratory distress syndrome (ARDS) and multiorgan failure, leading to a higher risk of death^3^ ^,^ ^4^.Due to the risk of viral contamination of patients and healthcare personnel, several surgical societies have recommended postponement of elective or deferable surgical procedures in the initial phases of COVID-19 pandemic^5^ ^-^ ^10^.Even when asymptomatic, COVID-19 positive patients were also shown to exhibit higher postoperative morbidity and mortality after general^11^ ^-^ ^15^ and gastrointestinal surgery^16^ ^-^ ^18^, leading several authorities to recommend systematic SARS-Cov2 screening by RT-PCR before surgery to lessen potential viral exposure to the surgical team and to decrease the incidence of patient’s postoperative complications^19^ ^-^ ^22^. Those policies have led to a sharp decline in surgical volumes of elective procedures worldwide with a gradual recovery of some but not all surgical proceduresin recent months toward baseline levels in most countries^23^ ^-^ ^25^.

The purpose of the present study was to evaluate the impact of COVID-19 pandemic in surgical volume and outcomes of abdominal surgery in high-risk patients requiring ICU admission in a tertiary care center in Brazil. 

## PATIENTS AND METHODS

All patients admitted to the Gastroenterology and Hepatology Unit of the Portuguese Hospital of Salvador, Bahia, Brazil after elective or emergency laparotomy from January 2017 to December 2022, were retrospectively evaluated except for those patients admitted after organ transplantation. This facility is an intensive gastrointestinal ICU dedicated to postoperative care of high-risk patients submitted to abdominal surgery. It remained a COVID free environment throughout the observation periodfor admission of high-risk surgical patients with a negative RT-PCR for SARS-CoV-2. The Portuguese Hospital of Salvador, Bahia is a non-profit private organization who remained active throughout the COVID-19 pandemic.

Data concerning demographics; year at admission; type and duration ofsurgery; surgical procedure; surgical wound classification; surgical team; comorbidity, according to Charlson Comorbidity Index (CCI) and presence of concurrent malignancy, Acute Physiology and Chronic Health Evaluation II (APACHE II) score, in the first 24 hours in the ICU; ICU and intrahospital length of stay (LOS) and mortality was retrospectively reviewed in two different periods of time between January 2017 and December 2019 and between January 2020 to December 2022, respectively, before (period I) and after (period II) the onset of COVID-19 pandemic. Patients in palliative care were excluded from the analysis.

Surgery was considered elective when it was scheduled or planned and urgent in the present of an acute event leading to admission in the emergency department and requiring surgery in the first 24 hours. Immediate need for surgery due to life threatening illnesses was required to characterize emergent surgical intervention. Surgical wound grades, as well as APACHE II score and CCI were classified and calculated as previously described^26^ ^-^ ^28^.

Surgical procedures were grouped as colorectal, pancreatic, gallbladder and biliary tract, gastric, liver, and other surgeries. Other surgeries included procedures with volumes lower than 100 interventions in the observation period including appendicectomy, splenectomy and bariatric, cytoreductive, esophageal, gynecologic or obstetrics, small bowel, retroperitonealand urologic surgeries and procedures when no organ resection was caried out in the presence for example of adhesions or hernia repair. Patients were followed until death or hospital discharge. The primary endpoint was in-hospital mortality.

The study was performed in accordance with principles of the Declaration of Helsinki andapproved by the Ethics Committee in Research of the Portuguese Hospital of Salvador, Bahia (reference number 26210819.5.0000.5029).

### Statistical analysis

Dichotomous variables are presented in text and tables as numbers and percentage and continuous variables were expressed as mean ± standard deviation (SD) or as median and interquartile range, respectively, whether the distribution was normal or skewed. Data concerning surgical procedures were compared using the chi-square test or Fisher’s test for categorical variables or Student’s t-test or the Mann-Whitney U test for continuous variables when appropriate. A p value <0,05 was considered significant. The software used for analysis was the Statistical Package for Social Sciences (SPSS Inc., Chicago, IL, EUA), version 14.0 for Windows. 

## RESULTS

**Figure 1:**
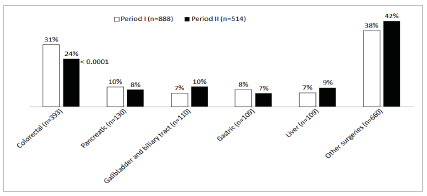
Number of surgical procedures admitted to the ICU.

One thousand four hundred and two high-risk patients (897 females, mean age 61,6+17,1 years) were admitted to the ICU between January 2017 and December 2022. Perioperative features of all patients are depicted in Table 1. Briefly, most procedures were elective (52,4%) with clean (15,5%) or clean-contaminated surgical wounds. The five most common interventions were colorectal (n=393), pancreatic (n=130), gallbladder and biliary tract (n=110), gastric (n=109) and liver (n=109) surgeries (Figure 1). Mean CCI and APACHE II at ICU admission were 4,5+2,9 and 10,1+5,6, respectively. One hundred twenty-five (8,9%) patients died, due to causes unrelated to SARS-CoV-2 infection. Most of the deaths were due to septic (n=120) and hypovolemic (n=3) shock andcardiovascular complications (n=2). The mean LOS in the ICU and in the hospital were 5,7 [2-6] and 11,9 [4-7] days, respectively (Table 1). 

**Table 1:** Demographics, clinical and postoperative features of surgical patients admitted to the ICU before and after the onset of COVID-19 pandemic.

	All patients (n=1402)	Period I (n=888)	Period II (n=514)	p
Age (years)	61,6 ± 17,1	60,06 ± 17,1	64,15 ± 16,7	0,21
Female gender	897 (64,0)	568 (63,4)	329 (63,3)	0,98
CCI (mean)	4,5 ± 2,9	4,35 ± 2,8	4,85 ± 3,0	0,002
Type of surgery				0,03
Elective	735 (52,4)	482 (54,3)	253 (49,2)	
Urgency	661 (47,1)	400 (45)	261(50,8)	
Emergency	6 (0,4)	6 (0,7)	0 (0)	
Wound classification				0,02
Clean	218 (15,5)	144 (16,2)	74 (14,4)	
Clean-Contaminated	971 (69,3)	593 (66,8)	378 (73,5)	
Contaminated	130 (9,3)	97 (10,9)	33 (6,4)	
Dirty/Infected	83 (5,9)	54 (6,1)	29 (5,9)	
Surgery duration (min)	236 ± 124	238 ± 123	233 ± 125	0,46
Postoperative APACHE II	10,1 ± 5,6	10,3 ± 5,7	9,7 ± 5,3	0,06
ICU LOS (days)	5,7 [2-6]	5,44	6,23	0,06
Hospital LOS (days)	11,9 [4-7]	12,03	11,64	0,70
Mortality	125 (8,9)	70 (7,9)	55 (10,7)	0,08

APACHE II: acute physiology and chronic health evaluation II, CCI: Charlson Comorbidity Index (CCI), ICU: intensive care unit, LOS: length of stay.

**Figure 2:**
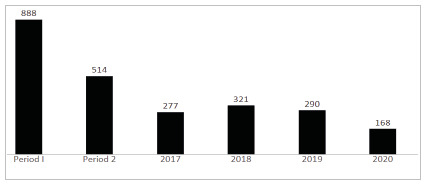
Number of admissions of high-risk surgical patients in the ICU according to year and period of time.

Comparison of demographics and perioperative features of those patients according to the observation period, before (period I) and after (period II) the onset of COVID-19 pandemic demonstrated a 42% reduction of surgical volume in the last period of observation without recovery in the following years after 2020 (Figure 2). Patients admitted postoperatively to the ICU in period II, when compared to period I had higher CCI (4,85+3,0 vs. 4,35+2,8 in period I, p=0,002). In addition, their surgical procedures were more frequently urgent (50,8% vs. 45% in period I, p=0,03), less frequently colorectal surgeries (24% vs. 31% in period I, p<0,0001) and more often other surgeries (42% vs. 38%, p<0,0001) (Table 1 and Figure 1). According to the adopted classification, there was an increase in clean contaminated surgical wounds after onset of the COVID-19 pandemic (73,4% vs. 66,8% in period I, p=0,02). No differences in demographics, duration of surgery and hospital LOS were observed. Postoperative APACHE II seemed to be lower in period II when compared to period I and ICU LOS longer when patients from period II were compared to their counterparts in period I, but the different was not statistically significant (Table 1). Likewise, mortality tended also to be higher in period II when compared to period I (10,7% vs. 7,9% in period I, p=0.08). 

## DISCUSSION

The present study demonstrated a 42% decrease in the volume of abdominal surgery of high-risk patients requiring ICU admission after the onset of COVID-19 pandemic without a major impact in surgical mortality. Our findings are consistent with several other authors, who demonstrated a decrease in the volume of several elective and emergency surgical procedures during the COVID-19 pandemic^23^ ^-^ ^25^ ^,^ ^29^ ^-^ ^34^,without a significant increase in mortalityin most^31^ ^,^ ^34^ ^-^ ^37^ but not all reports^32^ ^,^ ^38^ ^,^ ^39^. Higher mortality rates in some reports were attributed to higher frequencies of emergent/urgent procedures in sicker patients^39^ as well as lower availability of ICU beds during the initial waves of COVID-19 infection^40^. In contrast to some reports, showing gradual return of surgical volume to pre-pandemic levels in several countries and institutions^24^ ^,^ ^41^,our data demonstrated no increase in the volume of abdominal surgery in the last two years of observation. This is in accordance with several other reports demonstrating a large heterogeneity in surgical volume recovery even after the defervescence of COVID-19 pandemic in several parts of the world^23^ ^-^ ^25^.The reasons for this lack of surgical volume recovery, particularly due to colorectal surgery, observed in the present study as well as in several others^23^ ^-^ ^25^ is intriguing but may reflect the huge decrease in consultations and guidelines cancerscreening programs seen in the initial waves of COVID-19 pandemic. It is possible to speculate that many of those patients lost from healthcare due to social isolation measures may remainup to now undiagnosed or mayalternatively have progression to an unresectable stage of cancer afterwards. 

The purpose of this study was to evaluate the impact of COVID-19 pandemic in high-risk patients requiring abdominal,instead of general or cardiovascular surgery, who were referred to the ICU for postoperative care. When comparing the most frequent surgical procedures, a greater impact of COVID-19 pandemic was noted in the volume of colorectal procedures as highlighted by some other reports who additionally demonstrated a higher risk emergency presentation and more advanced stage of colorectal cancer at surgery after the onset of COVID-19 infection^39^ ^,^ ^42^ ^,43^.

In the same line of thinking, probably the colorectal cancer was the most affected disease in terms of early diagnosis when the tumor is still resectable. Colonoscopy is frequently indicated as screening exam for patients over forty-five or fifty years old, and it can detect asymptomatic small tumors. The fear of getting infected by COVID made the people stop doing colonoscopies during the pandemic time. Other cancers, like pancreas, liver and gallbladder were less affected by this issue, as they don’t have the same screening policy, what can explain the bigger impact in colorectal cancer with lower number even after the COVID period.

This increase in the frequency of emergent surgical procedures usually with more clean-contaminated wounds in those sicker patients with higher comorbidity are in accordance with the literature and may reflect initial worldwide recommendation for postponement of all unessential surgical procedures.The different abilities of public and private healthcare systems to recover from the healthcare disruptioncausedby COVID-19 pandemic and most importantly the delay in the public perception that elective surgery is nowadays as safe as before the COVID-19 pandemiccan be another reason for the reduced numbers of surgical procedures.

In summary COVID-19 pandemic was associated with a decrease in surgical volume of high-risk patients submitted to abdominal surgery without apparent recovery in following years. No influence of COVID-19 was noted in postoperative mortality.
